# Does bilateral rib augmentation with lamina bands enhance cranial anchoring in magnetically controlled growing rod treatment? Evaluation of complication rate

**DOI:** 10.1016/j.jor.2025.01.022

**Published:** 2025-01-15

**Authors:** Stefan Hemmer, Raphael Trefzer, Julian Deisenhofer, Lukas Baumann, Wojciech Pepke

**Affiliations:** a Clinic for Orthopaedics, Heidelberg University Hospital, Heidelberg, Germany; b Institute of Medical Biometry, University of Heidelberg, Heidelberg, Germany

**Keywords:** MCGR, Complication rate, Rib enlacement, Lamina band

## Abstract

**Background:**

Early-Onset Scoliosis (EOS) presents a challenging condition often associated with severe health implications if left untreated. Magnetically Controlled Growing Rods (MCGR) have revolutionized EOS management, offering less-invasive spinal correction and growth preservation. Despite these advancements, high complication rates (21 %–66 % in MCGR-treated patients), particularly cranial screw loosening, remain a concern.

**Objective:**

This study evaluates the complication rates associated with MCGR implantation in EOS and investigates the impact of bilateral rib augmentation with lamina bands on cranial anchoring stability.

**Methods:**

A retrospective review was conducted on 62 EOS patients treated with MCGR between 2013 and 2022. Clinical and radiographic data, including Cobb angles, sagittal alignment, and complication profiles, were analyzed. Patients with and without bilateral lamina band augmentation were compared to assess its impact on cranial screw loosening. Statistical analyses were performed using SPSS, with significance set at p < 0.05.

**Results:**

The cohort included 36 idiopathic (58 %) and 26 neuromuscular (42 %) scoliosis cases, with a mean age of 11.1 ± 2.4 years. Postoperative analysis demonstrated significant improvement in Cobb angle and apex deviation (p < 0.05). 13 of 62 patients (21 %) required an unplanned revision. Cranial screw loosening was reported in 13 cases (21 %), with 6 (10 %) of these requiring unplanned surgical revision. Bilateral rib augmentation with lamina bands showed a reduced rate of cranial screw loosening from 50 % to 10.9 % (OR = 8.2, p < 0.05). Thoracic kyphosis flattening post-implantation was linked to higher rates of cranial anchorage failure.

**Conclusion:**

MCGR treatment effectively corrects deformities and preserves spinal growth in EOS, but complications such as cranial screw loosening highlight the need for enhanced anchoring techniques. Rib augmentation with lamina bands significantly improves cranial stability and may reduce mechanical complications. Further research for optimizing surgical techniques and understanding biomechanical factors is crucial to improve outcomes.

## 1 Introduction

Juvenile and Early-Onset Scoliosis (EOS) is a complex spinal deformity that presents before the age of ten and is often associated with severe health implications, including restricted pulmonary development and increased mortality if left untreated.^1^^,^^2^ The traditional approach to managing EOS involves the use of Traditional Growing Rods (TGRs), which necessitate multiple surgeries to achieve spinal elongation, posing significant risks and burdens to patients and their families.^1^ For more than fifty years, growth-friendly surgical devices have been a central focus of technical advancements and scientific investigation.^3^^,^^4^ A significant breakthrough in the treatment of scoliosis in young children occurred with the development of magnetically controlled growing rods (MCGR).^5^ In 2014, the National Institute for Health and Care Excellence (NICE) in the UK endorsed the use of MCGRs for patients with EOS, citing their proven effectiveness and cost-efficiency.^6^ MCGRs represent a significant advancement in the treatment of EOS, offering a less invasive alternative to TGRs. MCGRs allow for non-surgical lengthening through an external remote control (ERC), thus reducing the frequency of surgical interventions and associated complications.^1^ Despite these advantages, MCGRs are not without their challenges. On the other side, in patients who still have to undergo relevant growth of the trunk, it is important to minimize fusion levels. Fewer anchoring in spinal constructs increase the risk of proximal and distal anchor failure,^7^ so a good balance must be aimed between preservation of growth and providing construct stability. Complication rates remain high, with common issues including implant failure, metallosis, and unplanned revision surgeries.^8^, ^9^, ^10^, ^11^, ^12^ The efficacy and safety of MCGRs have been the focus of numerous studies, with mixed outcomes reported. While some studies suggest that MCGRs provide comparable deformity correction and spinal growth to TGRs,^13^ others highlight significant complication rates that necessitate careful consideration and management.^2^ Despite advancements in the design and implementation of MCGR, the issue of cranial screw loosening remains a significant concern, warranting further investigation. The biomechanical changes introduced by MCGR implantation, particularly the loss of thoracic kyphosis (TK), are believed to play a critical role in possible loosening of the cranial anchorage. This correlation highlights the need for comprehensive research to better understand the mechanisms underlying screw loosening and to develop strategies that mitigate this complication.

This study aims to (1) evaluate the complication rates associated with the use of MCGRs in the treatment of EOS, providing a comprehensive analysis of patient outcomes and identifying potential risk factors for complications. Further, the aim (2) was to analyze if the bilateral rib augmentation with lamina bands enhance cranial anchoring in MCGR treatment. Through this investigation, we seek to contribute to the optimization of treatment protocols and improve patient care in the management of EOS.

## 2 Methods

### 2.1 Study cohort

**Fig. 1:**
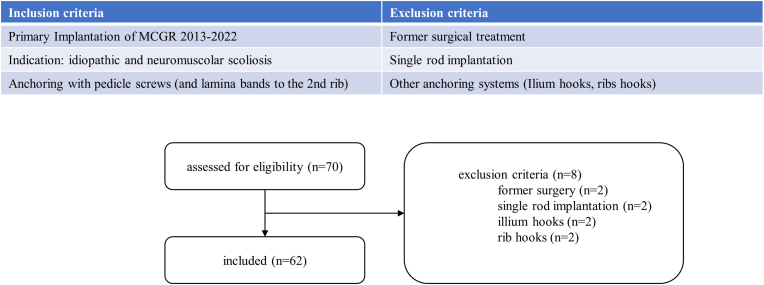
Diagram depicting the process of inclusion and exclusion criteria for the study population.

This retrospective study was conducted at a single center and focused on patients diagnosed with idiopathic and neuromuscular scoliosis before the age of 10 (Early-Onset Scoliosis, EOS) who underwent implantation of the MCGR system between 2013 and 2022. The study included patients with a Cobb angle greater than 45°, a Risser sign^14^ of 0, and a Sanders classification^15^ between 0 and 3 at the time of surgery. Inclusion criteria included the availability of full-spine EOS® radiographs or standard anterior-posterior and lateral radiographs obtained preoperatively, postoperatively, and during follow-up. Exclusion criteria included the use of alternative anchoring systems (ilium hooks or rib hooks), unilateral single rod implantation, and a history of prior spinal surgeries. The inclusion and exclusion process is illustrated in Fig. 1. Ethical approval for the study was granted by the ethics committee of the medical faculty of Heidelberg University (approval No. S-418/2022).

### 2.2 Surgical technique

**Fig. 2:**
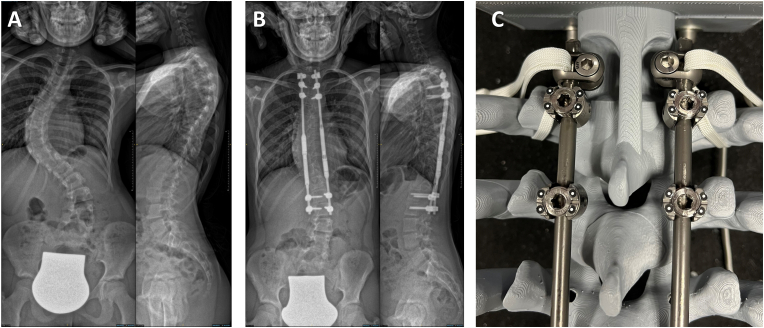
Anterior-posterior and lateral radiographs of a patient with idiopathic scoliosis (A) following MCGR implantation (B). Cranial anchoring with pedicle screws and lamina band encirclement around the second rib in a model (C).

The MCGR system features a magnetically controlled lengthening mechanism within a spinal distractible rod. In this study, all surgeries utilized a dual-rod construct with pedicle screw anchoring, customized to each patient's height and pedicle size. Preoperative standing X-rays were used to determine the vertebral levels for MCGR anchoring. For patients with neuromuscular scoliosis, the MCGRs were anchored from T2 to the ilium. The surgeries, performed under general anesthesia with neurological monitoring, involved two small posterior midline incisions. In idiopathic scoliosis cases, four pedicle screws were placed per incision, whereas neuromuscular scoliosis cases required six screws at both the upper thoracic and lumbopelvic incisions. The rods were inserted subfascially and connected to the screws. Standard MCGRs were placed on the concave side and offset MCGRs on the convex side to prevent magnetic interference during distractions. Pre- and postoperative radiographs, depicting the results before and after MCGR implantation, are shown in Fig. 2. Since 2018, additional cranial anchorage for the MCGR has been performed with laminar bands looped around the second rib. All patients remained free from postoperative neurological impairments.

### 2.3 Data collection and analysis

Radiographs were obtained using a stereoradiography device or standard imaging methods, capturing anterior-posterior and lateral views in an upright position. Standing patients were barefoot, with their hands on their clavicles, and instructed to look straight ahead. Neuromuscular scoliosis patients who were unable to stand were assessed in a sitting position. Baseline radiographs were used for measurements taken preoperatively, postoperatively, and prior to each outpatient ERC-induced rod lengthening session. Data were saved as DICOM files and analyzed using Surgimap® software. All measurements for coronal, sagittal and axial profile were performed by the second author (RT), a research fellow not involved in patient treatment. Apical vertebral rotation (AVR) of the main curve (Raimondi main curve) and the compensatory curve (Raimondi secondary curve) can be assessed using the Raimondi rotation angle. This method is considered a dependable approach for evaluating the degree of vertebral rotation as seen on standard upright spine radiographs.^16^ Clinical data were retrieved from patient records.

### 2.4 Statistical analysis

Statistical analyses were performed using SPSS® Version 25 (IBM®, USA). Data were presented as mean ± standard deviation. Comparisons of measurements taken preoperatively, postoperatively, and during follow-up were conducted using paired t-tests for comparison from pre-to postoperative values and unpaired *t*-test for intergroup comparison, with statistical significance set at p < 0.05. Cumulative revision-free implant survival was assessed according to Kaplan-Meier analysis with computation of 95 % confidence intervals (95 % CI). Odds ratios (OR) and absolute risk reduction (ARR) were calculated to evaluate the impact of band augmentation on pedicle screw loosening and dislocation rates.

## 3 Results

### 3.1 Global analysis

This study evaluated the outcomes of 62 patients who underwent MCGR implantation at our institution between 2013 and 2022, focusing on coronal, sagittal and axial profile correction, as well as postoperative complications. Due to the infeasibility of performing X-ray diagnostics in a vertical position for neurogenic scoliosis with severe cerebral palsy, the radiological data from two patients were not included in the analysis.

Idiopathic scoliosis was identified in 36 patients (58 % of the cohort), while neuromuscular scoliosis was diagnosed in 26 patients (42 %). The mean age at the time of surgery was 11.1 ± 2.4 years. Statistical comparisons between the idiopathic and neuromuscular scoliosis subgroups revealed no significant differences (p > 0.05) in terms of age at index surgery, BMI, postoperative follow-up duration, occurrence of MCGR lengthening failure, or measured parameters in the coronal and sagittal planes on X-rays. However, the number of instrumented segments was significantly higher in the neuromuscular group compared to the idiopathic group (14.9 ± 2.6 segments vs. 12.1 ± 1.3 segments; p < 0.001). Since the introduction of bilateral cranial anchoring augmentation using laminar bands wrapped around the second rib, a total of 46 patients have received this treatment. Of these, 26 patients (72 %) from to the idiopathic scoliosis group and 20 patients (77 %) from the neuromuscular group were treated with bilateral lamina band augmentation (Table 1).

**Table 1:** Patient characteristics from the medical records.

Parameter	idiopathic	neuromuscular	total
Number [n (%)]	36 (58 %)	26 (42 %)	62 (100 %)
Female [n (%)]	30 (83 %)	15 (58 %)	45 (73 %)
Age at surgery [mean (SD)]	11.6 (2.4)	10.5 (1.8)	11.1 (2.4)
BMI at surgery (m^2^/kg) [mean (SD)]	18.4 (3.9)	16.6 (4.5)	17.7 (4.3)
Band augmentation to 2nd rib [n (%)]	26 (72 %)	20 (77 %)	46 (74 %)
Instrumented segments [mean (SD)]	12.1 (1.3)	14.9 (2.6)	13.3 (2.4)
Follow-up [months (range)]	26 (1–103)	31.3 (4–91)	28.2 (1–103)

### 3.2 Analysis of adverse complications after MCGR implantation

**Fig. 3:**
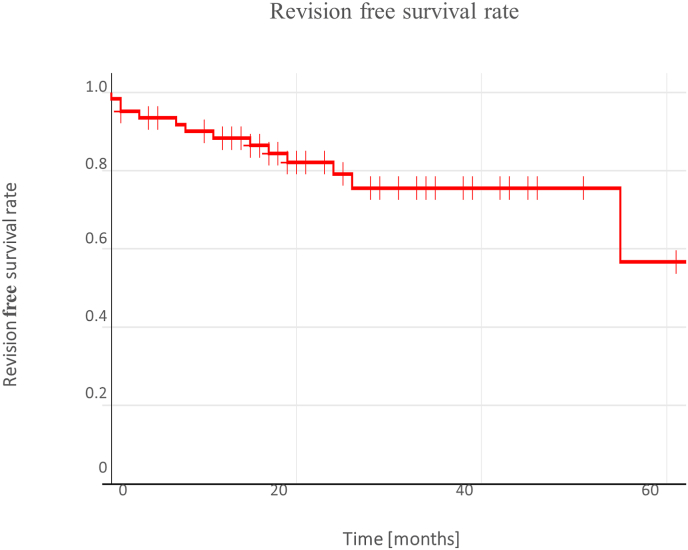
Diagram showing revision-free survival.

The analysis of the study population revealed that 13 out of 62 patients (21 %) required an unplanned revision. A total of 34 complications were identified in 26 patients. The most common complication was loosening of the implanted screws, which occurred in 18 patients (29 % of the cohort), with 6 patients (9.7 %) requiring an unplanned implant revision. Cranial screw loosening was observed in 13 patients (21 %), 6 of whom (9.7 %) exhibited dislocation of the screw-rod system; 2 patients (3.2 %) required an unplanned revision. Loosening of screws in the caudal anchorage was less frequent, occurring in 5 patients (8.1 %), with 1 patient (1.6 %) requiring revision. Infection, both early and late, was the second most common complication, affecting 6 patients (9.7 %). Of these, 5 patients (8.1 %) required an operative revision. Additional complications are listed in Table 2. An analysis of cumulative revision-free survival revealed rates of 0.88 (95 % CI: 0.77–0.94, number at risk: 50) after the first postoperative year, 0.79 (95 % CI: 0.65–0.88, number at risk: 28) after the second year, and 0.76 (95 % CI: 0.60–0.86, number at risk: 12) after the third year (Fig. 3).

**Table 2:** Presentation of recorded complications following MCGR implantation.

Complication	[n]	[%]	Revision [n]	[%]	Total
Cranial screws loosening (∗1 combined)	13	21.0	6	6.5	62
of which with screw dislocation	6	9.7	2	3.2	62
Caudal screws loosening (∗1 combined)	5	8.1	∗1	1.6	62
of which with screw dislocation	1	1.6	0	0	62
**Total screws loosening**	**18**	**29.0**	**6**	**9.7**	**62**
Early infection	2	3.2	2	3.2	62
Late infection	4	6.5	3	4.8	62
**Total infections**	**6**	**9.7**	**5**	**8.1**	**62**
Adjacent decompensation	4	6.5	0	0	62
Wound healing disorder	2	3.2	2	3.2	62
Decompensation of coronal alignment	2	3.2	0	0	62
Dislocation of screw-rod system	1	1.6	0	0	62
Serous thoracic effusion	1	1.6	0	0	62
**Total**	**34 (26 Pat.)**		**13**	**21.0**	**62**

### 3.3 Analysis of coronal and sagittal profile correction of the study group and impact of TK flattening on loosening of cranial anchoring

**Fig. 4:**
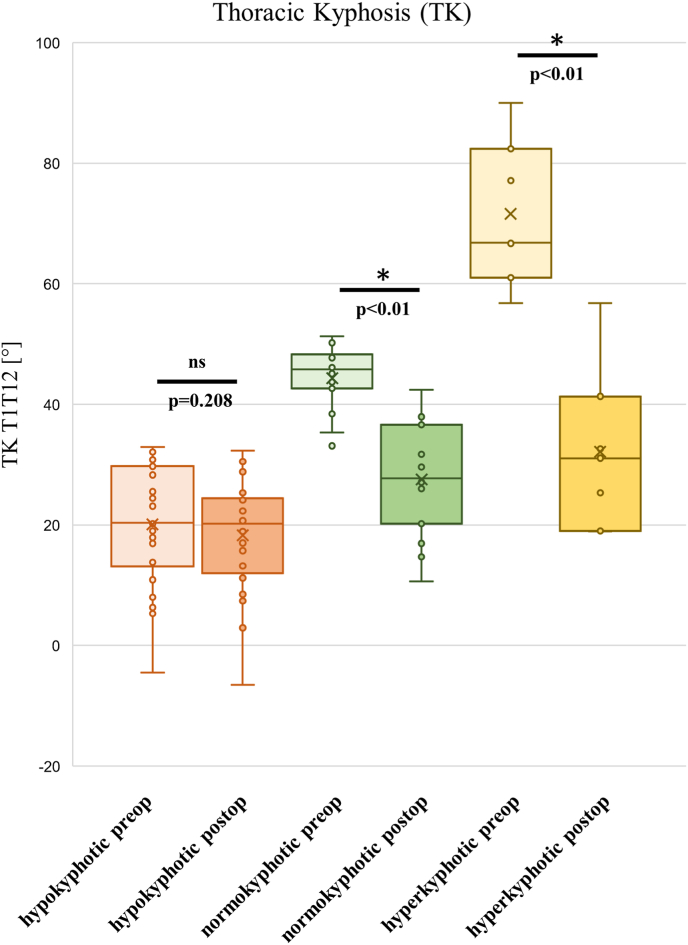
Diagram illustrating the changes in thoracic kyphosis (TK) from preoperative to postoperative in subgroups with thoracic hypokyphosis, normokyphosis, and hyperkyphosis. ∗ indicates a significant difference.

**Fig. 5:**
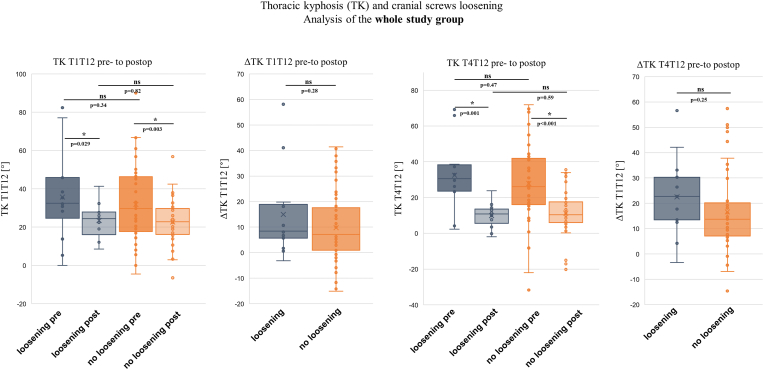
Diagrams showing the changes in TK-T1T12 and T-T4T12 from preoperative to postoperative in all patients with and without cranial screw loosening. ∗ indicates a significant difference.

Surgical treatment with MCGR implantation resulted in significant correction of the primary Cobb angle (68.4° ± 18.8°–38.0° ± 13.7°; p < 0.001 and secondary Cobb angle (44.1° ± 14.9°–28.0° ± 11.5°; p < 0.001). Similarly, apex deviation decreased significantly (51.7 ± 22.7 to 28.9 ± 15.1; p < 0.001), as did the Raimondi Index for the main curve (31.1 ± 10.2 to 22.8 ± 11.9; p < 0.001). Sagittal profile parameters for the cervical, lumbar, and pelvic regions showed no significant changes pre-to postoperatively (Table 3). However, analysis of the thoracic spine profile revealed significant postoperative changes. TK measured from T1 to T12 (TK-T1T12) was significantly reduced, flattening from 33.8° ± 19.7°–22.8° ± 10.8° (p < 0.001). Similarly, TK-T4T12 was significantly lower postoperatively (28.9° ± 20.9°–11.0° ± 10.7°; p < 0.001). Interestingly, kyphosis measured between T2 and T5 (TK-T2T5) increased postoperatively (9.0° ± 8.5°–15.7° ± 7.4°; p < 0.001). To investigate which patients experienced thoracic TK loss following MCGR implantation, the study cohort was stratified based on preoperative TK values according to established normative data.^17^ The hypokyphotic subgroup (n = 34) had TK < 33°, the normokyphotic subgroup (n = 19) ranged from 33° to 55°, and the hyperkyphotic subgroup (n = 7) had TK > 55°. Analysis revealed significant TK flattening pre-to postoperatively in the normokyphotic and hyperkyphotic subgroups (p < 0.05) (Table 4 and Fig. 4). Analysis of patients with cranial anchorage loosening (with and without laminar band augmentation around the second rib) compared to patients without loosening showed no significant differences in ΔTK-T1T12, and ΔTK-T4T12 (Fig. 5).

**Table 3:** Table presenting coronal and axial parameters before and after MCGR implantation. ∗ indicates a significant difference.

Coronal Profile	preop.		postop.	
Parameter	Mean	SD	Mean	SD
Cobb main curve	68.4	18.8	38.0∗	13.7
Cobb secondary curve	44.1	14.9	28.0∗	11.5
Coronar alignment	18.7	17.6	16.4	15.9
C7 plumb line	18.5	18.4	17.3	15.6
Apex deviation main curve	51.7	22.7	28.9∗	15.1
Apex deviation secondary curve	13.4	11.8	13.3	10.3
Raimondi main curve	31.1	10.2	22.8∗	11.9
Raimondi sec. curve	12.9	10.2	10.1	9.0

**Table 4:** Table stratifying the study population into hypokyphotic, normokyphotic, and hyperkyphotic subgroups regarding thoracic kyphosis (TK).

TK Group	preop	postop	n
	Mean (SD)	Mean (SD)	
hypokyphotic	20.1 (9.6)	18.3 (8.7)	34
normokyphotic	44.3 (4.8)	27.6 (8.7)	19
hyperkyphotic	71.5 (11.1)	32.1 (12.5)	7

### 3.4 Analysis of additive bilateral rib augmentation with lamina bands for cranial anchoring

**Fig. 6:**
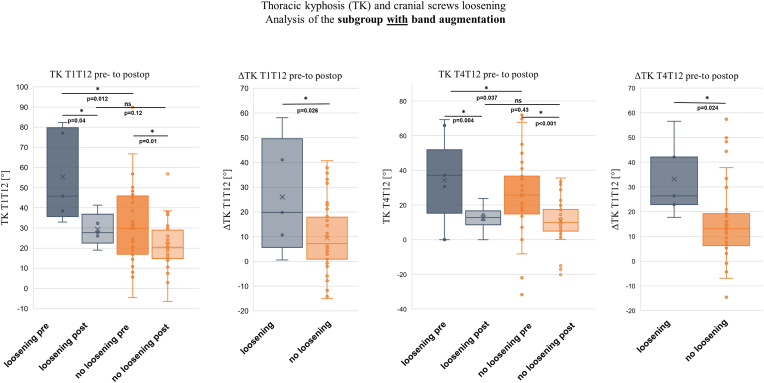
Diagrams showing the changes in TK-T1T12 and TK-T4T12 from preoperative to postoperative in bilateral rib band augmented-patients with and without cranial screw loosening. ∗ indicates a significant difference.

**Fig. 7:**
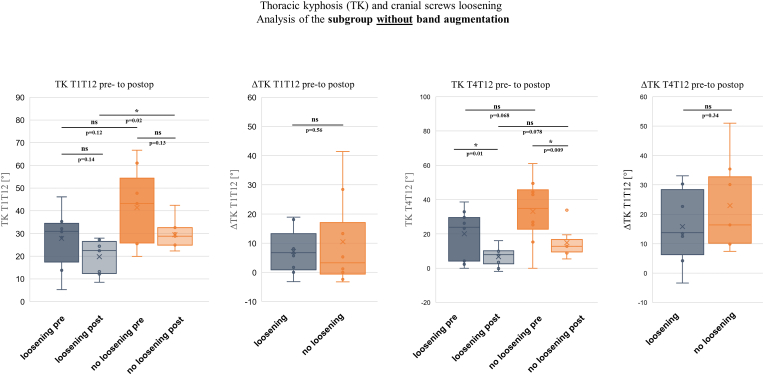
Diagrams showing the changes in TK-T1T12 and TK-T4T12 from preoperative to postoperative in patients with cranial screw anchoring (without rib augmentation), with and without cranial screw loosening. ∗ indicates a significant difference.

In this study population, cranial anchorage without rib augmentation using a lamina band was performed in 16 patients. Of these, 8 patients (50 %) experienced loosening of the cranial anchorage, with 3 patients (18.8 %) requiring revision. In contrast, 46 patients received bilateral lamina bands implanted around the second rib in addition to cranial screw anchorage. This group exhibited screw loosening in only 5 patients (10.9 %), with 3 patients (6.5 %) requiring revision. Statistical analysis of the contingency table was used to calculate the odds ratio (OR) and absolute risk reduction (ARR). The OR was 8.2, and the ARR was 39.1 % (Table 5). Interestingly, subgroup analysis of patients with cranial screws and additional lamina band augmentation revealed that those with anchorage loosening exhibited a significantly greater change in thoracic kyphosis (ΔTK-T1T12 and ΔTK-T4T12) compared to the group without anchorage loosening, as shown in Fig. 6. In contrast, subgroup analysis of patients with cranial screws but without bilateral rib augmentation showed no significant differences in ΔTK-T1T12 and ΔTK-T4T12 between the subgroup with screw loosening and the subgroup without anchorage loosening (Fig. 7).

**Table 5:** Table with probability calculations for cranial anchorage loosening.

Band augmentation of the 2nd Rib	Cranial screws loosening		Total
	yes	no	
yes	5 (10.9 %)	41	46 (100 %)
with revision	3 (6.5 %)	43	

no	8 (50 %)	8	16 (100 %)
with revision	3 (18.8 %)	13	

Odds Ratio:(8/8)/(5/41) = **8.2**.

ARR:50 %–10.9 % = **39.1 %**.

## 4 Discussion

The advent of MCGR has transformed the management of EOS by providing a minimally invasive method for spinal correction and lengthening,^18^^,^^19^ while also demonstrating reduced treatment costs.^20^ This study provides a comprehensive analysis of the complication rates and outcomes of MCGR in treating EOS patients. Our findings underscore the nuanced balance between achieving effective spinal deformity correction and mitigating the high complication rates associated with MCGR implantation.

The overall complication rate in our cohort aligns with previous studies, with 21 % of patients requiring unplanned revisions. Implant-related complications, including screw loosening and infection, remain significant challenges in the clinical use of MCGRs. These findings are consistent with previous literature highlighting complication rates ranging from 21 % to 66 % in MCGR-treated patients.^10^^,^^11^^,^^21^^,^^22^ Screw loosening, particularly at the cranial anchorage, was observed in 21 % of cases, with cranial dislocation occurring in 9.7 % of patients. This aligns with studies emphasizing the mechanical vulnerability of cranial anchor points.^9^^,^^23^ The addition of bilateral rib augmentation using lamina bands notably reduced cranial screw loosening rates to 10.9 % compared to 50 % in patients without augmentation. This improvement suggests that lamina bands provide enhanced biomechanical stability, a finding supported by previous research emphasizing the importance of anchoring stability in MCGR constructs.^10^^,^^24^

Our results demonstrate significant improvements in the primary and secondary Cobb angles, apex deviation, and the Raimondi Index, consistent with previous studies highlighting the efficacy of MCGRs in deformity correction.^11^^,^^21^^,^^24^ However, the flattening of TK, particularly in normokyphotic and hyperkyphotic subgroups, suggests a need for further optimization in surgical planning to preserve sagittal alignment. Studies have similarly reported that excessive TK flattening may compromise spinal biomechanics and increase the risk of mechanical complications.^9^^,^^11^ TK reduction through MCGR implantation is thought to alter the biomechanical load distribution along the spine, potentially increasing stress at the cranial and caudal anchor points.^25^ This increased stress might predispose screws to loosening and dislocation. In our study, patients who experienced significant postoperative flattening of TK, especially in normokyphotic and hyperkyphotic subgroups, demonstrated higher rates of cranial screw loosening. This aligns with findings from prior studies, which suggest that diminished TK compromises the natural curvature's ability to stabilize forces, leading to elevated mechanical demands on anchorage points.^10^^,^^11^ The use of lamina band augmentation around the second rib has shown promise in mitigating this effect by enhancing cranial anchorage stability, even in patients with pronounced TK flattening.^10^ These findings emphasize the need for careful preoperative planning and intraoperative techniques that preserve or restore thoracic kyphosis during MCGR implantation. Addressing this biomechanical factor could significantly reduce the risk of screw-related complications and improve the longevity of spinal constructs. The incorporation of lamina band augmentation appears to mitigate some of the mechanical complications associated with cranial anchorage, offering a potential pathway for improving construct stability. Additionally, addressing the causes of TK flattening through improved surgical techniques and preoperative planning could enhance long-term outcomes.

This study provides valuable insights into the outcomes and complications of MCGR implantation in EOS patients, but several limitations must be acknowledged. The retrospective single-center design introduces selection bias and limits generalizability. The small, heterogeneous patient cohort and varying follow-up durations may impact the consistency of long-term outcomes. Additionally, reliance on radiographic parameters overlooks functional and quality-of-life measures. While focusing on cranial screw loosening and lamina band augmentation, other potential complications or anchoring techniques were not comprehensively analyzed. Finally, biomechanical factors were discussed but not directly tested, and unaccounted confounding variables such as patient activity and comorbidities may have influenced the results. Future prospective, multicenter studies with larger and more homogeneous cohorts are needed to validate these findings and enhance their applicability.

The study highlights that MCGRs have revolutionized the treatment of EOS by enabling minimally invasive spinal correction and lengthening while reducing treatment costs. Despite demonstrating significant improvements in spinal deformity parameters, the high complication rate, including cranial screw loosening, remains a major challenge. Bilateral rib augmentation with lamina bands was found to enhance anchorage stability and reduce loosening rates, emphasizing the importance of biomechanical optimization and careful surgical planning.

## 5 Conclusion

MCGRs remain a promising yet imperfect solution for managing EOS. While they effectively correct spinal deformities and reduce the need for invasive procedures, their high complication rates necessitate ongoing innovation in implant design and surgical techniques. The addition of rib augmentation strategies shows promise in enhancing anchorage stability and reducing complications, providing a potential model for future advancements in growth-friendly spinal instrumentation.

## CRediT authorship contribution statement

**Stefan Hemmer:** Conceptualization, Methodology, Validation, Writing – original draft, Supervision. **Raphael Trefzer:** Software, Validation, Formal analysis, Investigation, Data curation, Visualization. **Julian Deisenhofer:** Formal analysis. **Lukas Baumann:** Formal analysis. **Wojciech Pepke:** Conceptualization, Methodology, Validation, Investigation, Resources, Writing – review & editing, Visualization, Supervision, Project administration, All authors have read and agreed to the published version of the manuscript.

## Informed consent statement

Patient consent was waived due to retrospective study design and permission by the Ethics Committee. Analyzed radiographs were obtained while daily praxis. No radiographs were obtained explicitly for this study.

## Data availability

The datasets generated during and analyzed during the current study are available from the corresponding author on reasonable request.

## Institutional review board statement

The study was conducted according to the guidelines of the Declaration of Helsinki, and approved by the Ethics Committee of University Heidelberg (permission No. S-418/2022).

## Declaration of generative AI and AI-assisted technologies in the writing process

No artificial intelligence assisted technology was used to carry out this study.

## Funding

This research did not receive any specific grant from funding agencies in the public, commercial, or not-for-profit sectors.
